# Supplementary material for: The C. elegans TspanC8 tetraspanin TSP-14 exhibits isoform-specific localization and function
Source: PLoS Genet. 2022 Jan 28;18(1):e1009936. doi: 10.1371/journal.pgen.1009936 (PMC8827444; doi:10.1371/journal.pgen.1009936)
Supplement: S1 Fig — (PDF) [file pgen.1009936.s001.pdf]

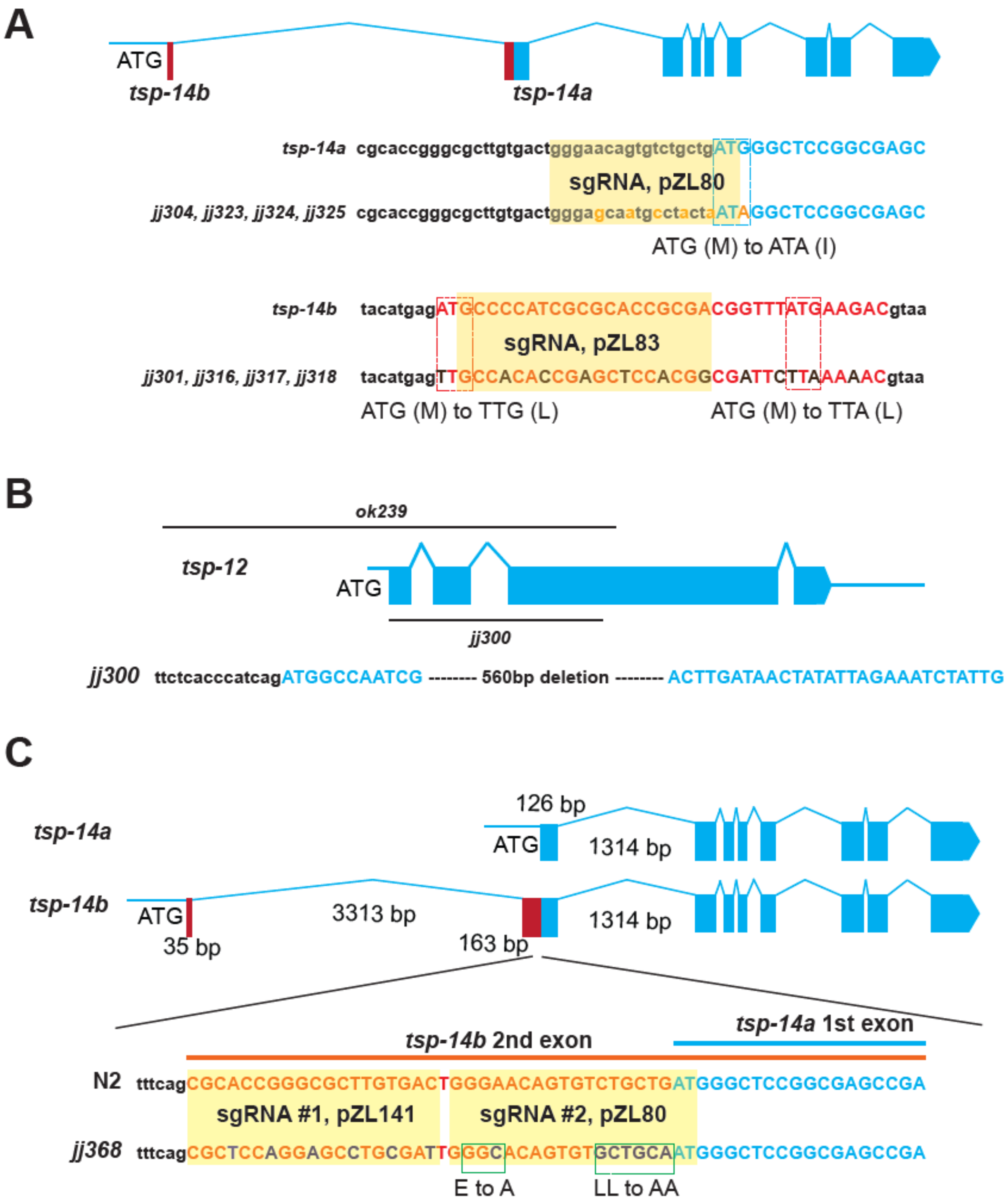
A
ATG
tsp-14b
tsp-14a
tsp-14a cgcaccgggcgcttgtgactgggaacagtgtctgctgATGGGCTCCGGCGAGC
sgRNA, pZL80
jj304, jj323, jj324, jj325 cgcaccgggcgcttgtgactgggagcaatgcctactaATAGGCTCCGGCGAGC
ATG (M) to ATA (I)
tsp-14b tacatgagATGCCCCATCGCGCACCGCGACGGTTTATGAAGACgtaa
sgRNA, pZL83
jj301, jj316, jj317, jj318 tacatgagTTGCCACACCGAGCTCCACGGCGATTCTTAAAAACgtaa
ATG (M) to TTG (L)
ATG (M) to TTA (L)
B
ok239
tsp-12
ATG
jj300
jj300 ttctcacccatcagATGGCCAATCG -------- 560bp deletion -------- ACTTGATAACTATATTAGAAATCTATTG
C
tsp-14a
126 bp
ATG
1314 bp
tsp-14b
ATG
35 bp
3313 bp
163 bp
1314 bp
tsp-14a 1st exon
tsp-14b 2nd exon
N2 tttcagCGCACCGGGCGCTTGTGACTGGGAACAGTGTCTGCTGATGGGCTCCGGCGAGCCGA
sgRNA #1, pZL141
sgRNA #2, pZL80
jj368 tttcagCGCTCCAGGAGCCTGCGATTGGGCACAGTGTGCTGCAATGGGCTCCGGCGAGCCGA
E to A
LL to AA

S1 Figure

S1 Figure. Information on the different mutations in *tsp-12* or *tsp-14* generated using CRISPR/Cas9. (A) *tsp-14* isoform specific knockout mutations. To specifically knock out *tsp-14a*, the start codon ATG (Met) of TSP-14A was mutated to ATA (Ile), to minimize significant impact on TSP-14B. To specifically knock out *tsp-14b*, the start codon ATG and another downstream, in-frame, ATG were mutated to TTG (Leu) and TTA (Leu), respectively. The sgRNA target site, as well as silent substitutions introduced to avoid cleavage of the repair templates by Cas9, are highlighted. **(B)** Schematics depicting the location of the two null mutations in *tsp-12*, *ok239* and *jj300*. The *jj300* allele was generated using CRISPR/Cas9, and the sequence of *jj300* is shown. **(C)** The EQCLL to AQCAA mutation in *tsp-14b*. Two sgRNAs used in this experiment. The sgRNA target sites, as well as silent substitutions introduced to avoid cleavage of the repair templates by Cas9, are highlighted. In all panels, coding sequences are in capital letters, while non-coding sequences are in lower cases.
