# Supplementary material for: The C. elegans TspanC8 tetraspanin TSP-14 exhibits isoform-specific localization and function
Source: PLoS Genet. 2022 Jan 28;18(1):e1009936. doi: 10.1371/journal.pgen.1009936 (PMC8827444; doi:10.1371/journal.pgen.1009936)
Supplement: S2 Fig — (PDF) [file pgen.1009936.s002.pdf]

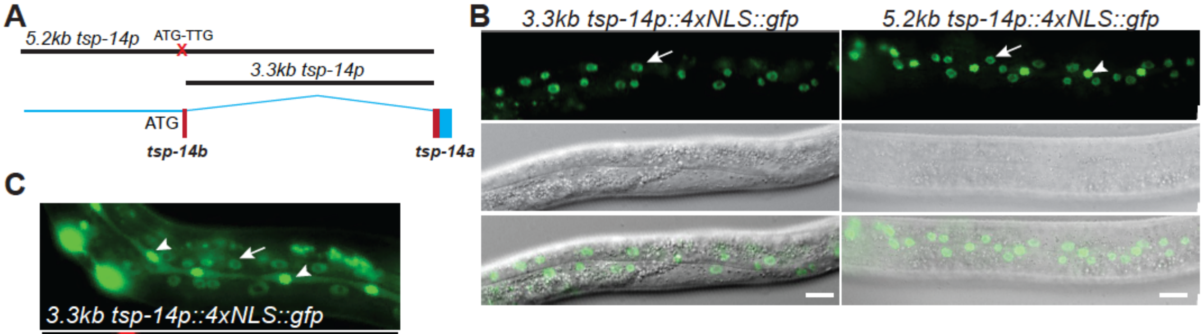

Genotype	Suppression	N
sma-9(cc604)	3%*	834
tsp-12(ok239); tsp-14(jj96) sma-9(cc604)	64.0%&	900
tsp-12(ok239); tsp-14(jj95) sma-9(cc604); jjEx4848[3.3kb tsp-14p::tsp-14a cDNA::tsp-14 3'UTR]	18.2%^	873
tsp-12(ok239); tsp-14(jj95) sma-9(cc604); jjEx4907[3.3kb tsp-14p::tsp-14b cDNA::tsp-14 3'UTR]	2.4%*	648

S2 Figure 2

S2 Figure. Transgenic reporters of *tsp-14*.

(A) Schematics of the two different *tsp-14* promoters used. They are 3.3kb and 5.2kb, respectively, upstream of the *tsp-14a* start codon. In constructs with the 5.2kb promoter, the start codon of *tsp-14b* was changed from ATG to TTG. **(B)** Images showing GFP expression in hypodermal cells in transgenic animals carrying either of the two *tsp-14* transcriptional reporters. Scale bar, 20 µm. **(C)** Images showing that the 3.3kb *tsp-14* promoter drives GFP reporter expression in hypodermal cells that also express a *sma-6p: 4xNLS::RFP* reporter. Notably, the 3.3kb *tsp-14p::4xNLS::GFP,* but not the *sma-6p: 4xNLS::RFP* reporter, is expressed in the seam cells. Arrows point to hypodermal cell nuclei. Arrowheads point to seam cell nucleus. Scale bar, 20 µm. **(D)** Table summarizing the penetrance of the Susm phenotype of *tsp-12(0); tsp-14(0)* animals with or without the transgene expressing *tsp-14a* or *tsp-14b*. *tsp-14(jj95)* and *tsp-14(jj96)* are both null alleles of *tsp-14* and behave identically, as reported previously (Wang et al., 2017). Groups marked with distinct symbols are significantly different from each other (*P*<0.001, in all cases when there is a significant difference), while groups with the same symbol are not. Tested using an ANOVA with a Tukey HSD (see Materials and Methods).
