# Supplementary material for: The C. elegans TspanC8 tetraspanin TSP-14 exhibits isoform-specific localization and function
Source: PLoS Genet. 2022 Jan 28;18(1):e1009936. doi: 10.1371/journal.pgen.1009936 (PMC8827444; doi:10.1371/journal.pgen.1009936)
Supplement: S3 Fig — (PDF) [file pgen.1009936.s003.pdf]

A

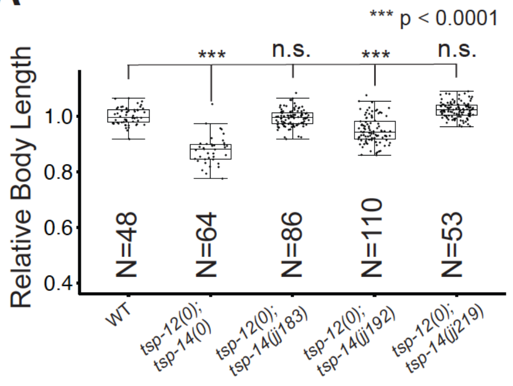

In panel A, *tsp-12(0): ok239*

B

Genotype	Suppression(%)	N
sma-9(cc604)	1.5±0.4*	476
tsp-12(ok239); sma-9(cc604)	13.1±1.8&	343
tsp-14(jj96) sma-9(cc604)	1.5±0.4*	502
tsp-12(ok239); tsp-14(jj96) sma-9(cc604)	64.0±1.0^	505
tsp-14(jj183) sma-9(cc604)	0.9±0.4*	616
tsp-12(ok239); tsp-14(jj183) sma-9(cc604)	36.0±0.9$	718
tsp-14(jj192) sma-9(cc604)	1.7±0.4*	424
tsp-12(ok239); tsp-14(jj192) sma-9(cc604)	6.9±0.3#	696

S3 Figure

S3 Figure. The functionality of *tsp-14* knock-ins in the *tsp-12(ok239)* background.

(A) Relative body lengths of synchronized L4 worms of various genotypes. The mean body length of wild-type worms is normalized to 1.0. For each double mutant, data were pooled from two independent isolates. The total number of worms measured for each genotype is at least 60. Tukey's HSD test following an ANOVA was used to test for differences between different genotypes. ***$P < 0.0001$. n.s., no significant difference. As shown, *tsp-12(0); tsp-14(jj192)* worms are slightly smaller than wild-type (WT) worms, but not as small as *tsp-12(0); tsp-14(0)* worms. **(B)** Table summarizing the results of the *sma-9(0)* suppression assay of various *tsp-14* knock-in alleles, in combination *tsp-12(ok239)*. Percentage of suppression was calculated by the number of worms with 1-2 M-derived CCs divided by the total number of worms scored. N represents the total number of worms counted. Data from two independent isolates were combined for each genotype. Groups marked with distinct symbols are significantly different from each other ($P<0.001$, in all cases when there is a significant difference), while groups with the same symbol are not. Tested using an ANOVA with a Tukey HSD (see Materials and Methods).
