# Supplementary material for: The C. elegans TspanC8 tetraspanin TSP-14 exhibits isoform-specific localization and function
Source: PLoS Genet. 2022 Jan 28;18(1):e1009936. doi: 10.1371/journal.pgen.1009936 (PMC8827444; doi:10.1371/journal.pgen.1009936)
Supplement: S1 Table — (PDF) [file pgen.1009936.s004.pdf]

Table S1. Strains genereated in this study.

Genotype	Strain Number
tsp-12 and *tsp-14* null mutations	
tsp-12(ok239)	LW3382
tsp-12(jj300)	LW5809
tsp-14(jj95)	LW3713
tsp-14(jj96)	LW3714
tsp-14a isoform specific knockout	
tsp-14(jj304: tsp-14a(ATG-ATA))	LW5836
tsp-14(jj323: tsp-14a(ATG-ATA))	LW5837
tsp-14(jj324: tsp-14a(ATG-ATA))	LW5838
tsp-14(jj325: tsp-14a(ATG-ATA))	LW5839
tsp-14b isoform specific knockout	
tsp-14(jj301: tsp-14b(ATG-TTG & ATG-TTA))	LW5787
tsp-14(jj316: tsp-14b(ATG-TTG & ATG-TTA))	LW5866
tsp-14(jj317: tsp-14b(ATG-TTG & ATG-TTA))	LW5867
tsp-14(jj318: tsp-14b(ATG-TTG & ATG-TTA))	LW5868
TSP-14B sorting signal mutation	
tsp-14(jj368: tsp-14b(EQCLL-AQCAA))	LW5998
tsp-14(jj192 jj368: gfp::3xFLAG::tsp-14b(EQCLL-AQCAA))	LW5743
tsp-14(jj368 jj378: tsp-14b(EQCLL-AQCAA)::gfp::3xFLAG)	LW6043
Endogeniously tagged proteins	
tsp-12(jj181: gfp::3xFLAG::tsp-12)	LW4453
tsp-14(jj219: tsp-14::gfp::3xFLAG)	LW4768
tsp-14(jj183: gfp::3xFLAG::tsp-14a)	LW4455
tsp-14(jj184: gfp::3xFLAG::tsp-14a)	LW4456
tsp-14(jj186: gfp::3xFLAG::tsp-14a)	LW4486
tsp-14(jj200: TagRFP::3xMyc::tsp-14a)	LW4574
tsp-14(jj201: TagRFP::3xMyc::tsp-14a)	LW4575
tsp-14(jj192: gfp::3xFLAG::tsp-14b)	LW4520
tsp-14(jj193: gfp::3xFLAG::tsp-14b)	LW4521
tsp-14(jj202: TagRFP::3xMyc::tsp-14b)	LW4566
tsp-14(jj326: TagRFP::3xMyc::tsp-14b)	LW4576
tsp-14(jj304 jj319: tsp-14::GFP::3xFLAG)	LW5895
tsp-14(jj304 jj327: tsp-14::GFP::3xFLAG)	LW5896
tsp-14(jj317 jj377: tsp-14::GFP::3xFLAG)	LW6023
Knock-in functionality	
gfp::3xFLAG::tsp-14a(jj183) sma-9(cc604); ccIs4438	
gfp::3xFLAG::tsp-14b(jj192) sma-9(cc604); ccIs4438	LW6067
tsp-12(ok239); gfp::3xFLAG::tsp-14a(jj183)	LW4506, LW4507
tsp-12(ok239); gfp::3xFLAG::tsp-14b(jj192)	LW4598, LW4599
tsp-12(ok239); gfp::3xFLAG::tsp-14a(jj183) sma-9(cc604); ccIs4438	LW5219, LW5220
tsp-12(ok239); gfp::3xFLAG::tsp-14b(jj192) sma-9(cc604); ccIs4438	LW5211
tsp-12(jj300); gfp::3xFLAG::tsp-14a(jj183)	LW6031, LW6032

tsp-12(jj300); gfp::3xFLAG::tsp-14b(jj192)	LW6037, LW6044
tsp-12(jj300); gfp::3xFLAG::tsp-14a(jj183) sma-9(cc604); ccIs4438	LW6038, LW6039
tsp-12(jj300); gfp::3xFLAG::tsp-14b(jj192) sma-9(cc604); ccIs4438	LW6063, LW6065
Knock-out functionality	
nT1[qIs51]/tsp-12(jj300); tsp-14(jj95)	LW5830, LW5831
nT1[qIs51]/tsp-12(jj300); tsp-14(jj304: tsp-14a(ATG-ATA))	LW5873
tsp-12(jj300); tsp-14(jj317: tsp-14b(ATG-TTG & ATG-TTA))	LW5950, LW5951
tsp-12(jj300); tsp-14(jj368: tsp-14b(EQCLL-AQCAA))	LW6006, LW6007
sma-9(cc604); ccIs4438	LW3711
tsp-12(jj300); ccIs4438	LW5991, LW5990
tsp-14(jj95) sma-9(cc604); ccIs4438	LW6021, LW6022
nT1[qIs51]/tsp-12(jj300); tsp-14(jj95) sma-9(cc604); ccIs4438	LW6048
tsp-14(jj304: tsp-14a(ATG-ATA)) sma-9(cc604); ccIs4438	LW5874, LW5875
nT1[qIs51]/tsp-12(jj300); tsp-14(jj304: tsp-14a(ATG-ATA)) sma-9(cc604); ccIs4438	LW5900, LW5901
tsp-14(jj317: tsp-14b(ATG-TTG & ATG-TTA)) sma-9(cc604); ccIs4438	LW5948, LW5926
tsp-12(jj300); tsp-14(jj317: tsp-14b(ATG-TTG & ATG-TTA)) sma-9(cc604); ccIs4438	LW5952, LW5953
tsp-14(jj368: tsp-14b(EQCLL-AQCAA)) sma-9(cc604); ccIs4438	LW6020
tsp-12(jj300); tsp-14(jj368: tsp-14b(EQCLL-AQCAA)) sma-9(cc604); ccIs4438	LW6045, LW6049
TSP-14A subcellular localization	
tsp-14(jj183: gfp::3xFLAG::tsp-14a); pwIs1149[snx-1p::TagRFP::rab-11]	LW6028
tsp-14(jj183: gfp::3xFLAG::tsp-14a); pwIs1058[snx-1p::TagRFP::eea-1]	LW6029
tsp-14(jj183: gfp::3xFLAG::tsp-14a); pwIs1116[snx-1p::TagRFP::rab-7]	LW6030, LW6040
TSP-12 and TSP-14A or TSP-14B co-localization	
tsp-12(jj195: TagRFP::3xMyc::tsp-12); tsp-14(jj192: gfp::3xFLAG::tsp-14b)	LW4634, LW4635
tsp-12(jj195: TagRFP::3xMyc::tsp-12); tsp-14(jj184: gfp::3xFLAG::tsp-14a)	LW4654, LW4655
tsp-12(jj181: gfp::3xFlag::tsp-12); tsp-14(jj200: TagRFP::3xMyc::tsp-14a)	LW4874
Extra-chromosomal array rescue experiments	
nT1[qIs51]/tsp-12(ok239); tsp-14(jj95) sma-9(cc604); arIs37(Secreted CC::gfp); jjEx4907[3.3kb Ptsp-14::tsp-14b cDNA+myo-2p::rfp]	LW4907
nT1[qIs51]/tsp-12(ok239); tsp-14(jj95) sma-9(cc604); arIs37(Secreted CC::gfp); jjEx4912[3.3kb Ptsp-14::tsp-14b cDNA+myo-2p::rfp]	LW4912
nT1[qIs51]/tsp-12(ok239); tsp-14(jj95) sma-9(cc604); arIs37(Secreted CC::gfp); jjEx4848[3.3kb Ptsp-14::tsp-14a cDNA, mec-7p::rfp]	LW4848
nT1[qIs51]/tsp-12(ok239); tsp-14(jj95) sma-9(cc604); arIs37(Secreted CC::gfp); jjEx4875[3.3kb Ptsp-14::tsp-14a cDNA, mec-7p::rfp]	LW4875
nT1[qIs51]/tsp-12(ok239); tsp-14(jj95) sma-9(cc604); arIs37(Secreted CC::gfp); jjEx4911[3.3kb Ptsp-14::tsp-14a cDNA, mec-7p::rfp]	LW4911
MosSCI insertion lines	
jjSi387[3.3kb tsp-14p::tsp-14a cDNA::tsp-14 3'UTR] I; tsp-14(jj95)	LW6224
jjSi388[3.3kb tsp-14p::tsp-14a cDNA::tsp-14 3'UTR] I; tsp-14(jj95)	LW6230
jjSi389[3.3kb tsp-14p::tsp-14b cDNA::tsp-14 3'UTR] I; tsp-14(jj95)	LW6225
jjSi390[3.3kb tsp-14p::tsp-14b cDNA::tsp-14 3'UTR] I; tsp-14(jj95)	LW6226
jjSi401[5.2kb tsp-14p::tsp-14a cDNA::tsp-14 3'UTR] I; tsp-14(jj95)	LW6244
jjSi402[5.2kb tsp-14p::tsp-14b cDNA::tsp-14 3'UTR] I; tsp-14(jj95)	LW6245
jjSi393[snx-1p::tsp-14a cDNA-gDNA chimera::gfp::3xflag::tbb-2 3'UTR] I; tsp-14(jj95)	LW6231
jjSi394[snx-1p::tsp-14a cDNA-gDNA chimera::gfp::3xflag::tbb-2 3'UTR] I; tsp-14(jj95)	LW6232
jjSi395[snx-1p::tsp-14b cDNA-gDNA chimera::gfp::3xflag::tbb-2 3'UTR] I; tsp-14(jj95)	LW6233
jjSi396[snx-1p::tsp-14b cDNA-gDNA chimera::gfp::3xflag::tbb-2 3'UTR] I; tsp-14(jj95)	LW6234

Functionality of MosSci lines	
jjSi388[3.3kb Ptsp-14::tsp-14a cDNA::tsp-14 3'UTR]; nT1[qIs51]/tsp-12(jj300); tsp-14(jj95)	LW6253, LW6254
jjSi390[3.3kb Ptsp-14::tsp-14b cDNA::tsp-14 3'UTR]; nT1[qIs51]/tsp-12(jj300); tsp-14(jj95)	LW6255, LW6256
jjSi401[5.2kb Ptsp-14::tsp-14a cDNA::tsp-14 3'UTR]; nT1[qIs51]/tsp-12(jj300); tsp-14(jj95)	LW6257, LW6258
jjSi402[5.2kb Ptsp-14::tsp-14b cDNA::tsp-14 3'UTR]; nT1[qIs51]/tsp-12(jj300); tsp-14(jj95)	LW6259, LW6260
jjSi393[snx-1p::tsp-14a cDNA-gDNA chimera::gfp::3xflag::tbb-2 3'UTR]; nT1[qIs51]/tsp-12(jj300); tsp-14(jj95)	LW6266, LW6267
jjSi395[snx-1p::tsp-14b cDNA-gDNA chimera::gfp::3xflag::tbb-2 3'UTR]; nT1[qIs51]/tsp-12(jj300); tsp-14(jj95)	LW6261, LW6262
jjSi388[3.3kb Ptsp-14::tsp-14a cDNA::tsp-14 3'UTR]; nT1[qIs51]/tsp-12(jj300); tsp-14(jj95) sma-9(cc604); ccIs4438	LW6249, LW6250
jjSi390[3.3kb Ptsp-14::tsp-14b cDNA::tsp-14 3'UTR]; nT1[qIs51]/tsp-12(jj300); tsp-14(jj95) sma-9(cc604); ccIs4438	LW6251, LW6252
jjSi401[5.2kb Ptsp-14::tsp-14a cDNA::tsp-14 3'UTR]; nT1[qIs51]/tsp-12(jj300); tsp-14(jj95) sma-9(cc604); ccIs4438	LW6263, LW6274
jjSi402[5.2kb Ptsp-14::tsp-14b cDNA::tsp-14 3'UTR]; nT1[qIs51]/tsp-12(jj300); tsp-14(jj95) sma-9(cc604); ccIs4438	LW6264, LW6265
jjSi393[snx-1p::tsp-14a cDNA-gDNA chimera::gfp::3xflag::tbb-2 3'UTR]; nT1[qIs51]/tsp-12(jj300); tsp-14(jj95) sma-9(cc604); ccIs4438	LW6272, LW6273
jjSi395[snx-1p::tsp-14b cDNA-gDNA chimera::gfp::3xflag::tbb-2 3'UTR]; nT1[qIs51]/tsp-12(jj300); tsp-14(jj95) sma-9(cc604); ccIs4438	LW6268, LW6269
Transcriptional reporters	
jjEx5717[5.2kb tsp-14p::4xNLS::gfp::unc-54 3'UTR + pRF4]	LW5717
jjEx5718[5.2kb tsp-14p::4xNLS::gfp::unc-54 3'UTR + pRF4]	LW5718
jjEx5719[5.2kb tsp-14p::4xNLS::gfp::unc-54 3'UTR + pRF4]	LW5719
jjEx5720[5.2kb tsp-14p::4xNLS::gfp::unc-54 3'UTR + pRF4]	LW5720
jjEx5721[5.2kb tsp-14p::4xNLS::gfp::unc-54 3'UTR + pRF4]	LW5721
jjEx5722[5.2kb tsp-14p::4xNLS::gfp::unc-54 3'UTR + pRF4]	LW5722
jjEx5723[5.2kb tsp-14p::4xNLS::gfp::unc-54 3'UTR + pRF4]	LW5723
jjEx5724[3.3kb tsp-14p::4xNLS::gfp::unc-54 3'UTR + pRF4]	LW5724
