# Supplementary material for: The C. elegans TspanC8 tetraspanin TSP-14 exhibits isoform-specific localization and function
Source: PLoS Genet. 2022 Jan 28;18(1):e1009936. doi: 10.1371/journal.pgen.1009936 (PMC8827444; doi:10.1371/journal.pgen.1009936)
Supplement: S2 Table — (PDF) [file pgen.1009936.s005.pdf]

Table S2. Oligonucleotides and plasmids used in this study.

Oligonucleotides for genotyping mutants and knock-ins

tsp-12(ok239) genotyping:

JKL1223 (TGTGCTGCCATGTGGCTTTC, F)
LW47 (GTTTCGAGCAGTTTTACGCCACC, R)
JKL1399 (CGACGATTGGGATAGAAACACCTATTTTTC, F)
or
LW47 (GTTTCGAGCAGTTTTACGCCACC, R)
LW49 (GGAATCAACGGAGCCGACGAT, R)
LW50 (AAGTTTCGGCAGACATCCTTCCG, F)

tsp-12(jj300) genotyping:

ZL670 (CGTCGGCCAGAACATCATC, F)
ZL671 (TTTCCGATTCACCACCAGC, R)
ZL672 (AAACGAACACCGTGCCC, F)
or
ZL374 (CCCTCTGCTGTGCTCTGTGTTGC, F)
ZL375 (CTGAAGGGGAACATTTCTTTCCTGG, R)
ZL539 (TTTTGTGACCGGCATAGGCTGTG, F)

tsp-14(jj95, jj96) genotyping:

ZL178 (CGCTTGTGACTGGGAACA, R)
ZL179 (GACACACCGAGATACACTGAAA, F)
ZL180 (CAGAAGGACACGCGCTTTAT, F)

tsp-14a(jj304, jj323, jj324, 325) genotyping:

ZL518 (GCCGTCACTCATAACACCCATTC, F)
ZL675 (GACTGGGAGCAATGCCTACTAATAG, F)
ZL676 (GGGAATTTGCAACTTTGACC, R)
followed by sequencing using ZL518

tsp-14b(jj301, jj316, jj317, 318) genotyping:

ZL635 (TTCCTGAAGCTAGGCAGC, F)
ZL636 (CATCTTGGTCCACGAACACC, R)
ZL641 (CCACGGCGATTCTTAAAAAC, F)
followed by sequencing using ZL635

tsp-14b(EQCLL-AQCAA, jj368) genotyping:

ZL693 (CGCGGTTCACTGATTTCGC, F)
ZL694 (CTGTGGCGGCTTTGTTTGG, R)
ZL723 (GAGCCTGCGATTGGGC, F)
followed by sequencing using ZL694 or
ZL518 (GCCGTCACTCATAACACCCATTC, F)
ZL519 (CGTGGTTTGCGTTGCTTGTGTG, R)
ZL588 (CTGCGATTGGGCCCAATGCGCTG, F)
followed by sequencing using ZL518

gfp::3×flag::tsp-14b(jj192, jj193, 202, jj326) genotyping:

ZL349 (CGCCGGAATCACCCACGGAATGG, F)
ZL428 (CGTCTTCATAAAACGTCGCGGTG, R)
ZL537 (CGACTTCCTGAAGCTAGGCAGC, F)

tsp-14::gfp::3×flag(jj219, jj319, jj327, jj377, jj378) genotyping:

ZL349 (CGCCGGAATCACCCACGGAATGG, F)
ZL502 (CGCGTTCGAGACAGAAAGATTTGG, F)
ZL513 (GCGACAACGACTCGAACACCTCC, R)

gfp::3×flag::tsp-12(jj181) genotyping:

ZL431 (GGAGAATCTGTACTTTCAATCCGG, F)
ZL374 (CCCTCTGCTGTGCTCTGTGTTGC, R)
ZL375 (CTGAAGGGGAACATTTCTTTCCTGG, F)

gfp::3×flag::tsp-14a(jj183, jj184, jj186) genotyping:

ZL516 (CAAGGAGAATCTGTACTTTCAATCCGG, F)
ZL376 (CGCGTCGGCTCGCCGGAGCC, R)
ZL377 (CGTTATCCTGTTGGTCACGCAGG, F)

tagRFP::3×Myc::tsp-14a(jj200, jj201) genotyping:

ZL430 (TTATACGAAGTTATTTTCAGGGAGCCGG, F)
ZL376 (CGCGTCGGCTCGCCGGAGCC, R)
ZL377 (CGTTATCCTGTTGGTCACGCAGG, F)

sma-9(cc604) sequencing:

MLF69 (CGCAACAAGTTCATTCTCCA, F)
MLF70 (CTTGGCTAAGATCCCATGCT, R)
followed by sequencing using MLF69

tagRFP::3×Myc::tsp-12(jj195) genotyping:

ZL430 (TTATACGAAGTTATTTTCAGGGAGCCGG, F)
ZL374 (CCCTCTGCTGTGCTCTGTGTTGC, F)
ZL375 (CTGAAGGGGAACATTTCTTTCCTGG, R)

Oligonucleotides for sgRNA plasmid construction and/or repair template used in CRISPR/Cas9-mediated genome editing.

For pZL58 (*tsp-12* knockout #1 sgRNA):

ZL255 (TCTTGACTCGCTCAACGGGCAAAA)
ZL256 (AAACTTTTGCCCGTTGAGCGAGTC)

For pZL79 (*tsp-12* knockout #2 sgRNA):

ZL334 (TCTTGATGGCCAATCGACGACAGC)
ZL335 (AAACGCTGTCGTCGATTGGCCATC)

For pZL8 (*tsp-14* knockout #1 sgRNA):

LW1 (CGGGAATTCCTCCAAGAACTCGTACAAAAATGCTCT)
ZL28 (ACGCGAGCTCGCGCGGTGGTCAAACATTTAGATTTGCAATTCAATTATATAG)
LW2 (CGGAAGCTTCACAGCCGACTATGTTTGGCGT)
ZL27 (GACCACCGCGCGAGCTCGCGTGTTTTAGAGCTAGAAATAGCAAGTTA)

For pZL 9(*tsp-14* knockout #2 sgRNA):

LW1 (CGGGAATTCCTCCAAGAACTCGTACAAAAATGCTCT)
ZL30 (CGGATGCCTTCAGCCGCTTCAAACATTTAGATTTGCAATTCAATTATATAG)
LW2 (CGGAAGCTTCACAGCCGACTATGTTTGGCGT)
ZL27 (GAAGCGGCTGAAGGCATCCGGTTTTAGAGCTAGAAATAGCAAGTTA)

For pZL80 (*tsp-14a* specific knockout #1 sgRNA):

ZL336 (TCTTGGGGAACAGTGTCTGCTGAT)
ZL337 (AAACATCAGCAGACACTGTTCCCC)

tsp-14a specific knockout repair oligo

ZL639 (CACATTCTTTCAGCGCACCGGGCGCTTGTGACTGGGAGCAATGC
CTACTAATAGGCTCCGGCGAGCCGACGCGAGCTCGCGCGGTGGTCTC)

For pZL83 (*tsp-14b* specific knockout #2 sgRNA):
ZL342 (TCTTGGCCCCATCGCGCACCGCGA)
ZL343 (AAACTCGCGGTGCGCGATGGGGCC)

tsp-14b specific knockout repair oligo
ZL638 (TTTTGTCTGAAACCATTCTGAAATAATCTACATGAGTTGCCACA
CCGAGCTCCACGGCGATTCTTAAAAACGTAAGTGTTGTTTTGTA
GAGGGAAAAGAGACACAC)

For pZL59 (sgRNA, C-terminally tagged TSP-14 with *jj304*, *jj317* or *jj368* mutations.)
ZL257 (TCTTGGTACTGCAGCAAACCGATT)
Zl258 (AAACAATCGGTTTGCTGCAGTACC)

For pZL66 (Repair template, C-terminally tagged TSP-14 with *jj304*, *jj317* or *jj368* mutations.)
ZL291 (ACGTTGTAAAACGACGGCCAGTCGCCGGCAATTAAATCTGCCCG
CTCCTCCTG)
ZL292 (CATCGATGCTCCTGAGGCTCCCGATGCTCCGGACTTGGACTTCTG
TGGGACGAGATCAGTTTGCTGCAGTACCCATTGATGC)
ZL293 (CGTGATTACAAGGATGACGATGACAAGAGATAAATGTTATAGTAA
ATGATATTCAAATTTAAC)
ZL294 (GGAAACAGCTATGACCATGTTATCGATTTCGTTAAACGGATCAAG
CAAGCAAAATC)

For pZL80 (sgRNA, *tsp-14b* sorting signal mutated (EQCLL – AQCAA))
ZL336 (TCTTGGGGAACAGTGTCTGCTGAT)
ZL337 (AAACATCAGCAGACACTGTTCCCC)

For pZL141 (Repair template, *tsp-14b* sorting signal mutated (EQCLL – AQCAA))
ZL585 (TCTTGCGCACCGGGCGCTTGTGAC)
ZL586 (AAACGTCACAAGCGCCCGGTGCGC)

For pZL60 (*gfp::3×flag::tsp-12* or *tagRFP::3×Myc::tsp-12* knock-in #1 sgRNA):
ZL267 (TCTTGCCACTTCTCACCCATCAGA)
ZL268 (AAACTCTGATGGGTGAGAAGTGGC)

For pZL79 (*gfp::3×flag::tsp-12* or *tagRFP::3×Myc::tsp-12* knock-in #2 sgRNA):
ZL334(TCTTGATGGCCAATCGACGACAGC)
ZL335(AAACGCTGTCGTCGATTGGCCATC)

For pZL64 (*gfp::3×flag*::*tsp-12* knock-in repair template):
ZL283 (ACGTTGTAAAACGACGGCCAGTCGCCGGCAACCTCCCGTCGCTT
TGTTCA)
ZL284 (TCCAGTGAACAATTCTTCTCCTTTACTCATCTGATGGGTGAGAAGT
GGCCGA)
ZL285 (CGTGATTACAAGGATGACGATGACAAGAGAATGGCCAATCGACGA
CAGCCAGTGCAACACAGAGCACAGCAGAG)
ZL286 (TCACACAGGAAACAGCTATGACCATGTTATGCTGAGAATTCGGCGA
TGAGC)

For pZL134 (*tagRFP::3×Myc*::*tsp-12* knock-in repair template):
ZL283 (ACGTTGTAAAACGACGGCCAGTCGCCGGCAACCTCCCGTCGCTT
TGTTCA)
ZL286 (TCACACAGGAAACAGCTATGACCATGTTATGCTGAGAATTCGGCGA

TGAGC)
ZL358 (CTTGATGAGCTCCTCTCCCTTGGAGACCATCTGATGGGTGAGAAGT GGCCGA)
ZL359 (GAGCAGAAGTTGATCAGCGAGGAAGACTTGATGGCCAATCGACGA CAGCCAGTGCAACACAGAGCACAGCAGAG)

For pZL80 (*gfp::3×flag::tsp-14a* or *tagRFP::3×Myc::tsp-14a* knock-in sgRNA):
ZL336 (TCTTGGGGAACAGTGTCTGCTGAT)
ZL337 (AAACATCAGCAGACACTGTTCCCC)

For pZL81 (*gfp::3×flag::tsp-14a* or *tagRFP::3×Myc::tsp-14a* knock-in repair template):
ZL338 (ACGTTGTAAAACGACGGCCAGTCGCCGGCAACTGAGG GTGCTTGAGAGTGGC)
ZL339 (TCCAGTGAACAATTCTTCTCCTTTACTCATCAGCAGACAC TGTTCCCAGTCAC)
ZL340 (CGTGATTACAAGGATGACGATGACAAGAGAATGGGCTCC GGCGAGCC)
ZL341 (TCACACAGGAAACAGCTATGACCATGTTATACTGTACCGTGA CTCGGCGC)

For pZL83 (*gfp::3×flag::tsp-14a* or *tagRFP::3×Myc::tsp-14a* knock-in sgRNA):
ZL342 (TCTTGGCCCCATCGCGCACCGCGA)
ZL343 (AAACTCGCGGTGCGCGATGGGGCC)

For pZL84 (*gfp::3×flag::tsp-14a* or *tagRFP::3×Myc::tsp-14a* knock-in repair template):
ZL344 (ACGTTGTAAAACGACGGCCAGTCGCCGGCACCGTTTGCCTAAAT TAGATTTCGCCAC)
ZL345 (TCCAGTGAACAATTCTTCTCCTTTACTCATCTCATGTAGATTATTT CAGAATGGTTTCAG)
ZL346 (CGTGATTACAAGGATGACGATGACAAGAGAATGCCCCATCGCGCA CCGCGACGTTTTATGAAGACGTAAGTGTTGTTTTGTAGAGG)
ZL347 (TCACACAGGAAACAGCTATGACCATGTTATGTTGCATGTTTGAAACCC TCAAATGTTG)

Oligonucleotides for sgRNA plasmid construction and/or repair template used in CRISPR/Cas9-mediated Mos single copy insertion (MosSCI) with chromosome I ttTi4348.

For pZL170(*3.3kb tsp-14p::tsp-14a cDNA::tsp-14 3'UTR*, MosSCI repair template):
ZL735 (tgtaaaacgacggccagtgcGTAAGTGTTGTTTTGTAGAG)
ZL736 (ggaaacagctatgaccatgcGACAGTTTAAAACAGTAAATTTCAG)

For pZL171(*3.3kb tsp-14p::tsp-14b cDNA::tsp-14 3'UTR*, MosSCI repair template):
ZL735 (tgtaaaacgacggccagtgcGTAAGTGTTGTTTTGTAGAG)
ZL736 (ggaaacagctatgaccatgcGACAGTTTAAAACAGTAAATTTCAG)

For pZL172(*5.2kb tsp-14p::tsp-14a cDNA::tsp-14 3'UTR*, MosSCI repair template):
ZL739 (tgtaaaacgacggccagtgcACTCGAGAAATTAGTTGC)
ZL740 (caacacttacGTCTTCATAAACCGTCGC)
ZL741 (ttatgaagacGTAAGTGTTGTTTTGTAGAG)
ZL736 (ggaaacagctatgaccatgcGACAGTTTAAAACAGTAAATTTCAG)

For pZL173(*5.2kb tsp-14p::tsp-14b cDNA::tsp-14 3'UTR*, MosSCI repair template):
ZL739 (tgtaaaacgacggccagtgcACTCGAGAAATTAGTTGC)
ZL740 (caacacttacGTCTTCATAAACCGTCGC)
ZL741 (ttatgaagacGTAAGTGTTGTTTTGTAGAG)

ZL736 (ggaaacagctatgaccatgcGACAGTTTAAAACAGTAAATTTCAG)
For pJKL1226(*snx-1p::tsp-14a cDNA-gDNA chimera::gfp::3xflag::tbb-2 3'UTR*, MosSCI repair template):<br>JKL1940 (TCGATCATCCtgtaaaacgacggccagtgcGAAGGAATTGTCGAGCTTTTCA GTTTCTTTTAC)<br>JKL1938 (GTCGGCTCGCCGGAGCCCATCCGGTTACCTTCCAGGTGGAC)<br>JKL1939 (GTCCACCTGGAAGGTAACCGGATGGGCTCCGGCGAGCCGAC)<br>JKL1941 (gacactttttgggagttcaaggcttacACATTTGAGCAAGAAAATATTGTCGC GCAAG)<br>JKL1942 (CTTGCGCGACAATATTTTCTTGCTCAAATGTgtaagccttgaactcccaaaaagtgtc)<br>JKL1943 (tgcttgaaaggattttgcatttatcTTAAGTTATCTCTTGTCATCGTCATC CTTGTAATC)
For pJKL1227(*snx-1p::tsp-14b cDNA-gDNA chimera::gfp::3xflag::tbb-2 3'UTR*, MosSCI repair template):<br>JKL1940 (TCGATCATCCtgtaaaacgacggccagtgcGAAGGAATTGTCGAGCTTTTCA GTTTCTTTTAC)<br>JKL1944 (CGCGGTGCGCGATGGGGCATCCGGTTACCTTCCAGGTGGAC)<br>JKL1945 (GTCCACCTGGAAGGTAACCGGATGCCCCATCGCGCACCGCG)<br>JKL1941 (gacactttttgggagttcaaggcttacACATTTGAGCAAGAAAATATTGTCGC GCAAG)<br>JKL1942 (CTTGCGCGACAATATTTTCTTGCTCAAATGTgtaagccttgaactcccaaaaagtgtc)<br>JKL1943 (tgcttgaaaggattttgcatttatcTTAAGTTATCTCTTGTCATCGTCATC CTTGTAATC)
**Primers used to confirm genomic DNA sequence for knock-ins and knockouts**
tsp-12(jj300) sequencing:<br>ZL670 (CGTCGGCCAGAACATCATC)<br>ZL672 (AAACGAACACCGTGCCC)<br>followed by sequencing using ZL670
tsp-12(jj300) sequencing:<br>ZL375 (CTGAAGGGGAACATTTCTTTCCTGG, R)<br>ZL539 (TTTTGTGACCGGCATAGGCTGTG, F)<br>followed by sequencing using ZL539
tagRFP::3×Myc::tsp-12(jj257, jj258)<br>ZL531(CTGATTTCCGATTCACCACCAGCG, F)<br>ZL375 (CTGAAGGGGAACATTTCTTTCCTGG, R)<br>followed by sequencing using ZL531
tsp-14::gfp::3×flag(jj218, jj219)<br>ZL536 (GGAAAGAGAGATGGGGTGGGG, F)<br>ZL513 (GCGACAACGACTCGAACACCTCC, R)<br>followed by sequencing using ZL536
tsp-14::tagRFP::3×Myc(jj265, jj266)<br>ZL513 (GCGACAACGACTCGAACACCTCC, R)<br>ZL536 (GGAAAGAGAGATGGGGTGGGG, F)<br>followed by sequencing using ZL536
tsp-14a(jj304, jj323, jj324, 325) sequencing:<br>ZL693 (CGCGGTTCACTGATTTCGC, F)

ZL694 (CTGTGGCGGCTTTGTTTGG, R)<br>followed by sequencing using ZL693
tsp-14b(jj301, jj316, jj317, 318) sequencing:<br>ZL691 (GCCACCCTCATCCACTGC, F)<br>ZL692 (CAATTACAATTTTCTGTGGGGTTCG, R)<br>followed by sequencing using ZL691
**Primers used to confirm genotype of different MosSCI knock-ins**
jjSi388, jjSi390, jjSi401, jjSi402 genotyping:<br>ZL746 (GCAGAAATACCTCCCTGTCAATTCC)<br>ZL747 (TGAGCGTATCTATCAAGTCCTTGTCTC)<br>ZL748 (ACGGATTTAGTGACAGTTTCTTGGG)
jjSi393, jjSi395 genotyping:<br>ZL746 (GCAGAAATACCTCCCTGTCAATTCC)<br>ZL747 (TGAGCGTATCTATCAAGTCCTTGTCTC)<br>ZL763 (GACGTGGGGGGAATTGGTGG)
**Primers used to confirm genomic DNA of different MosSCI knock-ins**
jjSi388, jjSi390, jjSi393, jjSi395, jjSi401, jjSi402 genomic DNA sequencing:<br>ZL749 (GCGGAATACGAATTGGGAGACG)<br>ZL750 (TGAAATCTGAAGCACTGCCGC)<br>Sequencing with both ZL749 and ZL750.<br>ZL377 (CGTTATCCTGTTGGTCACGCAGG)<br>ZL751 (GCGATGGCTAGAATTCCGGAC)<br>Sequencing with both ZL377 and ZL751.<br>ZL752 (GTCCGGAATTCTAGCCATCGC)<br>ZL747 (TGAGCGTATCTATCAAGTCCTTGTCTC)<br>Sequencing with both ZL752 and ZL747.
