# Supplementary material for: The C. elegans TspanC8 tetraspanin TSP-14 exhibits isoform-specific localization and function
Source: PLoS Genet. 2022 Jan 28;18(1):e1009936. doi: 10.1371/journal.pgen.1009936 (PMC8827444; doi:10.1371/journal.pgen.1009936)
Supplement: Attachment — Submitted filename: Liu et al-response to reviewers-12-23-21.pdf [file pgen.1009936.s009.pdf]

Cornell University
Molecular Biology & Genetics
439/441 Biotechnology Building
Ithaca, NY 14853, USA
Website: www.mbg.cornell.edu

Dr. Jun (Kelly) Liu
Professor
Phone: (607) 254-8942 (Office)/254-1237 (Lab)
Fax: (607) 255-6249/255-2428
E-mail: JL53@CORNELL.EDU

December 23, 2021

Professors Greenwald and Copenhaver
PLoS Genetics Editorial Office
PLOS
1160 Battery Street,
Koshland Building East, Suite 100
San Francisco, CA 94111

Dear Dr. Greenwald and Dr. Copenhaver,

Enclosed please find our revised manuscript **PGENETICS-D-21-01478** entitled "**The *C. elegans* TspanC8 tetraspanin TSP-14 exhibits isoform-specific localization and function**". We very much appreciate the careful reviews and the positive responses from the two reviewers on our manuscript. We have revised our manuscript based on the referees' comments and suggestions (detailed below).

We also greatly appreciate the suggestion made by Dr. Greenwald on the addition of a model figure to summarize how the different isoforms may be contributing to different pathways or processes based on the genetic analysis. We have in fact spent much time trying to construct a meaningful model based on all the available data. In the end, we decided against it because we do not have enough molecular information on the different partners of the different tetraspanin proteins in different cell types, let alone in different subcellular compartments. We feel that a model without such information is not informative or may be even misleading. Despite the lack of a model, we hope that you will find the revised version appropriate for acceptance and publication.

We thank you for your time and consideration.

Sincerely,

Jun (Kelly) Liu
Professor
Molecular Biology and Genetics
Cornell University

Reviewer #1:

1) Line 113. The statement that "tsp-14b has an additional 163bp segment upstream of the shared 126bp sequences" seems incorrect. tsp-14b has an additional 72bp, thereby adding 24 aa.

We apologize for the lack of clarity in our original statement. Our original description was referring to the two transcripts, *tsp-14a* and *tsp-14b*, with additional sequences in the 5' untranslated region of *tsp-14b*. Thus it is longer than the 24aa (72bp) coding sequence. We have modified the text on page 6 of the revised manuscript to clearly indicate that the two transcripts share the same 3' end but with different 5' ends.

2) Line 130. Maybe state explicitly that the various Met mutations would prevent any in-frame product being made through a downstream ATG.

We thank the reviewer for this suggestion. We have made the changes in the revised manuscript on page 7.

3) Figure 2. 2A. Also show the extent of jj95 deletion? 2C¬–M. These images were very hard to see, in part because the resolution was so poor. The ROI (the vulva) could be made much larger to fill most of the frame. In addition, if the images contained lethal embryos, that wasn't clear. Although I generally accept statements that 100% or 0% of the double mutants were/weren't Vul etc., but it would still be good to put a number on that (n>100?). Was VPC induction zero or 100% or something in between? 2P. Significance values (Fisher's etc) should be likely be provided when making conclusions about differences in penetrance.

We have modified Figure 1 and added information to show the extent of the *jj95* deletion, which deletes most of the coding region of both *tsp-14a* and *tsp-14b*. The legend for Figure 1 has also been updated accordingly.

We are sorry about the low resolution of the images shown. The figures were downsized when we originally submitted the manuscript to prevent the file being enormously large. We have included high resolution images in this submission.

We have made the vulval region bigger as suggested by the reviewer. We did observe the vulva phenotype in a large number of animals (n>100). But we did not carefully follow vulva development to precisely determine whether the defect is due to lack of VPC induction or something in between. We have modified the text on page 7 of the revised manuscript to reflect this. We have also added the number of animals or embryos examined in the figure legend for Figure 2.

As described in materials and methods, we have done statistical analysis comparing the penetrance of the Susm phenotype in each genetic background. We have now added the information in the revised legends for Figures 2, 3, 4, 8, S2 and S3.

4) Figure 2 and others. In my view, it would be helpful to consistently substitute intuitive/informative names for the generated variants rather than using jj-alleles (e.g., in panel 2P). This was done to some extent (e.g., 2B), but doing so more consistently throughout out the paper would make the reading and interpretation easier. For example, with expression studies it might be useful to refer to the three GFP tags as TSP-14AB(CT), TSP-14AB(NT), TSP-14B(NT) etc.

We thank the reviewer's suggestion! We have made the recommended changes throughout the manuscript, including figures and figure legends.

5) Figure 2B and others. Is the statement that the error bars are 95% CI correct? More typically box and whiskers bars represent the range (not including outliers), which is what these appear to be. Moreover, if they were indeed 95% CIs, then the reported low p-values for a number of the comparisons would not be possible it seems.

We thank the reviewer for spotting this error of ours. When we initially worked on this manuscript and its figures, we presented all the body size data using bar graphs with error bars. But we decided in the end to use the box plot format to present the data so that readers can see the range of variations more accurately. But in that process, we forgot to change the figure legends. We sincerely apologize for this. We have made corrections in the legends for all the figures that have box plots (Fig 2, 3, 4, 8 and S3).

6) Line 140. Does EMB lethality refer to maternal effect lethality (otherwise could not score other larval and adult parameters like Susm)?

Yes, as stated on page 7, the EMB in this manuscript refers to maternal effect embryonic lethality. To make it easier for the readers, in particular non-worm people, we decided to use EMB rather the conventional MEL.

7) Figures 2, 3, and S2. If I understood the data correctly, it seems hard to easily rectify the results in Figures 2, 3, and S2 regarding the functions of the two isoforms. With respect to body length, Fig 2B indicates a specific role for TSP-14A with no detected function for TSP-14B. In contrast, Fig 3C indicates at least as strong a role for TSP-14B in body length as TSP-14A. With respect to Susm, in Fig 2P the tsp-12; tsp-14B double suppressed to only 37% versus 80% for tsp-12; tsp-14 (double null). But the data in Fig 3B indicate suppression of "tsp-12; tsp-14B" (jjSi388 or jjSi401) to be 80%, equal to that of the double null. Moreover, in Fig 3B the "tsp-12; tsp-14A" (jjSi390 or jjSi402) clearly suppresses to a strong extent (~50%), which is well above tsp-12 alone, in contrast to what was observed in Fig 2P. Lastly, Fig S2D indicates that the "tsp-12; tsp-14B" (Ex4848) and the "tsp-12; tsp-14A" (Ex4907) are either very weakly or not suppressed. Because extrachromosomal arrays were used in this experiment, it seems possible that over- or mis-expression of the isoforms could account for the observed rescue. On the one hand I give credit to the researchers for being thorough and carrying out these parallel approaches. And it's potentially interesting that they got different results. But it also seems difficult to make any clean conclusions and some of these discrepancies weren't directly addressed.

We really appreciate the careful observations made by this reviewer. We agree that with respect to body size, the results from knock-in and knock-out studies are not as clear-cut as we expected, and may even be contradictory. We admit our lack of insight on the mechanistic basis of this. We commented this in the Discussion on pages 17-18, which begins with "The roles of TSP-14A and TSP-14B in body size regulation appear more complicated." Regarding the Susm phenotype though, all our data are consistent with TSP-14B being the major TSP-14 isoform that functions redundantly with TSP-12 in patterning the postembryonic mesoderm, with TSP-14A also playing a role in this process. Again, we discussed this on pages 16-17 in the Discussion.

As recognized by the reviewer, we have tried multiple parallel approaches (knock-ins, knock-outs, extrachromosomal transgenic arrays, single copy transgene insertions) to address the functions of each isoform. However, each approach has its limitations. For example, both extrachromosomal and single copy

transgenic methods suffer possible over-, under-, or mis-expression problems, while knock-ins or knock-outs could have unforeseen consequences on the endogenous locus. We are planning additional mechanistic studies that are beyond the scope of this manuscript, which hopefully will clarify the roles of each of the TSP-14 isoform in *C. elegans* development.

8) Figure 4. 4D. Though intuitive, it might be helpful to have arrows marking each isoform. 4C. The Susm effect of tsp-14(jj183) is very minor – is it statistically significant? Although the logic for these experiments were clear enough, the overall findings in this figure (the tags don't strongly affect function) seem quite minor and more in line with a supplemental-type figure.

We have added white and blue dots (white: TSP-14B, blue: TSP-14A) marking each of the isoforms on the western blot in the revised Figure 4D.

We have also added the results of the statistical analysis to Figure 4C, and all other figures showing the Susm data.

We have decided not to move this figure to supplemental materials, because 1) we believe that it is important to show functional characterization of all the different tagged forms of TSP-14; 2) Figure 4C shows that *tsp-14(jj192)* (TSP-14B(NT)), which has an increased level of TSP-14B, can partially suppress the Susm phenotype of *tsp-12(0).* This second point is one of the major pieces of evidence supporting a redundant role of TSP-14B and TSP-12 in postembryonic mesoderm patterning.

9) Figure 5 and associated text. The DIC panels for A', C', F', G', and K' didn't show up on my PDF for some reason. I also did not see any 5N. Regarding the statement that "TSP-14A is primarily localized within intracellular vesicles, while TSP-14B is mainly localized on the cell surface," it may be hard for most readers to recognize this from the images. Perhaps providing a z-stack location in the panel or supplemental z-stack movies that scan through the worm, would helpful. With respect to differences in the intensity of GFP expression, this might best be represented with a simple quantification of intensities.

We are sorry that the DIC images didn't show up for the reviewer. But we are not sure why this is the case.

Indeed, there is no panel 5N in this figure. We have re-labeled the panels in the revised figure. We thank the reviewer for spotting this.

We agree with the reviewer that supplemental z-stack movies will help better illustrate the localization of TSP-14A and TSP-14B. We have added three supplemental movies, as movies S1, S2 and S3, showing z-stacks of TSP-14A(NT), TSP-14B(NT), and TSP-14AB(CT) in hypodermal cells.

We believe that because TSP-14B is localized to the plasma membrane, while TSP-14A is intracellularly localized as dispersed puncta, a simple quantification of fluorescence intensities does not accurately reflect the differences in the expression levels of TSP-14A and TSP-14B. Instead, the western blot shown in Figure 4D shows much more clearly that TSP-14B is expressed at a significantly lower level than TSP-14A.

10) Figure 6 and associated text. I was slightly confused about why the text referred to the images as TSP-14A. I realize that TSP-14A expression is much higher in the epidermis than TSP-14B and is somewhat less apical, but in principle the GFP-tagged strains would mark both TSP-14A and TSP-14B. Perhaps simply referring to it as "TSP-14AB(NT)" and making the above points would be most accurate? Along those lines, was there a reason not to use some of the other generated variants that would definitively mark A (or B)

isoforms only? 6H. Very minor, but the Mander's value (GFP::TSP-12 that overlaps with TagRFP::TSP-14A (the forth bar) for this seems rather high based on the representative images (E,F,G).

The reviewer is correct in that, in principle, both TSP-14A and TSP-14B should be tagged at their N-termini in *jj183* animals. However, since we never observed any band representing TSP-14B on western blots (Figure 4D), or detected any cell surface localization of the fluorescent signal in *jj183* animals, we believe that the GFP insertion may have either disrupted the splicing or affected the expression of TSP-14B. We have commented this more explicitly on page 9 of the revised manuscript. Thus, we have labeled the panels in Figure 5 as TSP-14A being N-terminally tagged in either *jj183* or *jj200* animals.

In general, we have found that for the same protein, TagRFP signals are always fainter than the corresponding GFP signals. Furthermore, tagged TSP-14A (both GFP and TagRFP) always has much brighter signals than the corresponding GFP or TagRFP-tagged TSP-12. Thus, as described in the legend for Figure 6, "PCC indicates the correlation of GFP::TSP-14A with TagRFP::TSP-12 or TagRFP::TSP-14A with GFP::TSP-12. MOC indicates the fraction of TagRFP::TSP-12 that overlaps with GFP::TSP-14A (the second bar), or the fraction of GFP::TSP-12 that overlaps with TagRFP::TSP-14A (the forth bar).", the MOC value in the second bar is higher than the PCC value in the first bar. The values for the third and fourth bars are indeed similar, as we re-analyzed the data, and the Mander's values shown in Figure 6 are correct. We believe that the perceived difference between the images and the quantifications shown might be due to differences in the intensity of the fluorescence signals, as described above. To help alleviate this problem, we have decided to slightly increase the brightness of the TagRFP-TSP-14A signal shown in panel E of the revised Figure 5.

11) Figure 7 and associated text. The text leads with the statement that "TSP-14B::GFP::3xFLAG in tsp-14a null (jj304 jj319) animals is localized to the basolateral surface of vulval and hypodermal cells." But the data in Fig 7 refer specifically to intestine and pharynx, which was something of a disconnect. Was normal TSP-14B expression detected at the BL membrane in these tissues and is there a reason not to report findings for vulval and hypodermal cells? Also, was there an a priori reason to suspect that TSP-14B localization would depend on TSP-14A?

Unfortunately, the single copy transgene using the *snx-1* promoter and the *tbb-2* 3'UTR did not give us strong expression in the hypodermal and vulval epithelial cells. We believe that this is due to the *tbb-2* 3'UTR (which is optimal for germline transgene expression) that we used for transgene expression, because the *snx-1* promoter should be active in hypodermal and vulval cells. Fortunately, we were able to detect fluorescence signal in intestinal cells, which are also polarized, allowing us to draw conclusions about the basolateral membrane localization of TSP-14B. We have discussed this on page 13 of the revised manuscript, and have also added the reference (Merritt et al., 2008) describing the original finding.

As discussed above, because knock-ins or knock-outs could have unforeseen consequences on the endogenous locus, even though we observed basolateral membrane localization of TSP-14B(CT) in *tsp-14a* null (*jj304 jj319*) animals, we wanted to use an independent and parallel method to determine if TSP-14B alone can localize to the basolateral surface in a *tsp-14(0)* null background, thus the single copy transgene experiment.

12) Figure 8. Again, you may want to provide supporting statistical data when comparing suppression levels of different alleles (also mentioned above).

As discussed in our response to point 9) above, we believe that the western blot shown in Figure 4D is a much clearer reflection of the expression levels of different tagged forms of TSP-14, including in the AQCAA mutants (*jj322 jj192* and *jj368 jj378*).

Reviewer #2:

1). Page 11, "TSP-14A colocalizes with both the early endosome marker RAB-5 and the late endosome marker RAB-7, but shows little co-localization with the recycling endosome marker RAB-11 (Figure 6I-6R)."

--Given that RAB-7 is also associated with lysosomes in addition to late endosomes, the co-localization of TSP-14A and RAB-7 might indicate that TSP-14A is also enriched on lysosomes.

We thank the reviewer's suggestion. We have made the recommended changes on page 12 of the revised manuscript.

2). Fig 7B and Page 12 claim that TSP-14B::GFP is localized to the basolateral membrane of the intestinal cells.

--From Fig 7B, it looks that the GFP signal is strongly enriched on the luminal side of the intestine, which is supposed to be the apical side of intestinal cells. Although the basolateral membrane localized GFP is also visible, the signal intensity is weaker than that observed on the luminal side.

We respectively disagree with the reviewer's assessment that Figure 7B is showing the apical localization of TSP-14B. We have imaged numerous worms during the course of our studies on TSP-12 and TSP-14, and have never observed any apical plasma membrane localization of either protein in the intestinal epithelial cells. Perhaps re-looking at the following images will help a bit. Below, we have marked the faint background shadowy intestinal lumen using white arrows. They clearly do not have any TSP-14B signal.

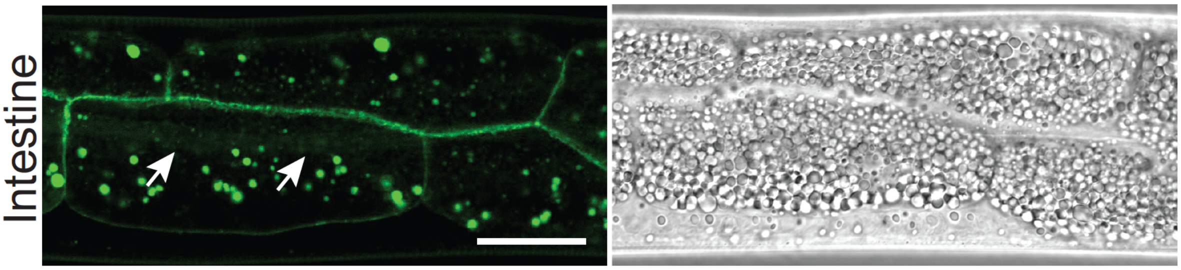

3). page 13, "We found that the GFP tagged AQCAA mutant TSP-14B protein in jj322 jj192 worms becomes localized to the apical side of the developing vulva (compare Figure 8C vs 8F) and in intracellular vesicles of hypodermal cells (Figure 8D vs. 8G), just like TSP-14A".

--Related to point 2 above, after studying Figure 8 C/F and D/G, what I observe is that the AQCAA mutations of the proposed di-leucine motif of TSP-14B primarily change the GFP localization from the plasma membrane to intracellular vesicles rather than from the basolateral to apical membrane. One possible function of the EQCLL motif might be to retain TSP-14B on the plasma membrane by slowing down the entry of PM-localized TSP-14B into the endocytic recycling pathway. Is this idea testable?

Yes, the reviewer is correct that TSP-14B with the AQCAA mutations is localized to intracellular vesicles rather than the basolateral plasma membrane (as shown in the hypodermal cells which are extremely thin). However, in the developing vulva, it is very clear that WT TSP-14B is only localized to the basolateral membrane (Figure 8C), while TSP-14B with the AQCAA mutations is not only localized to intracellular vesicles, but also only on the apical side. To help illustrate that point, we direct the reviewer to Figure 5O-5V, where we have included corresponding DIC images of the vulva, showing clearly which is the apical side and which is the basolateral side.

The idea that "the function of the EQCLL motif might be to retain TSP-14B on the plasma membrane by slowing down the entry of PM-localized TSP-14B into the endocytic recycling pathway" is an interesting one and could be tested in the future. However, we believe that it is beyond the scope of this manuscript.

4). Regarding the extra 24 amino acids of TSP-14B, which contains the di-leucine motif, is the di-leucine motif the only part that is important for the function of TSP-14B? When tsb-14b cDNA bearing the "AQCAA" mutations is expressed under the promoter that drives tsp-14a expression, given that TSB-14B(AQCAA) is intracellular localized, like TSP-14A, can it replace tsp-14a function?

This is a great question that we are interested in addressing too. However, to address this question, we need to 1) define the regulatory element(s) regulating *tsp-14a* expression; 2) rule out the possibility that TSP-14A level is regulated post-transcriptionally (because we observed much higher levels of TSP-14A than TSP-14B on western blots). At present, we do not have any insight into either one of the above questions. We feel that experimentally addressing this question is beyond the scope of the current work.

5). Page 16, "Three lines of evidence suggest that TSP-14B, but not TSP-14A, is the major player that functions redundantly with TSP-12 in patterning the postembryonic mesoderm".

--As reported here, TSP-14B is plasma membrane localized, yet TSP-12 is known to be localized to endosomes, it is hard to reason how TSP-14B functions in a molecular activity redundant to TSP-12. It is more likely that the pathway includes TSP-14B and the pathway includes TSP-12 display redundant functions.

We have previously reported that TSP-12 is both localized to the membrane, in particular, basolateral membrane, of polarized epithelial cells, and in various endosomal vesicles (Wang et al, 2017 PLoS Genetics). As shown in this manuscript, TSP-14B is localized to the basolateral membrane. It is therefore highly likely that TSP-12 and TSP-14B share redundant molecular activities at the membrane. However, we cannot rule out the possibility that TSP-14B-mediated pathway shares functional redundancy with TSP-12-mediated pathway. We hope that our future work on these two proteins will help address this question.
